# Failed Zone II Flexor Tendon Repair: What's Next?

**Published:** 2016-06-01

**Authors:** Xingchen Li, Alexis L. Parcells, Ramazi O. Datiashvili

**Affiliations:** Division of Plastic Surgery, Department of Surgery, Rutgers New Jersey Medical School, Newark

**Keywords:** flexor tendon injury, zone II injury, flexor tendon rupture, zone II rupture, failed zone II repair

## DESCRIPTION

A 51-year-old woman presented to the emergency department after sustaining a transverse volar laceration over the middle phalanx of her right small finger. On physical examination, the patient was unable to flex her finger at the proximal or distal interphalangeal joint.

## QUESTIONS

1. **How would you classify the aforementioned injury?**
2. **What are the challenges involved in treating this acute injury?**
3. **What is the importance of hand therapy and follow-up after tendon repairs?**
4. **What are options for reconstruction after a failed primary tendon repair?**

## DISCUSSION

Flexor tendon injuries are categorized into 5 zones defined by anatomical landmarks. Zone 1 consists of injuries in the space distal to the flexor digitorum superficialis insertion. Subsequent zones are demarcated at their proximal margins by the A1 pulley (zone II) as seen in the aforementioned injury, the distal edge of the carpal tunnel (zone III), proximal edge of the carpal tunnel (zone IV), and proximal to the carpal tunnel (zone V). This classification helps guide prognosis and management of flexor tendon injuries, as the anatomical characteristics of each dictate the ease of repair and the required stringency of postoperative follow-up.^1^

Zone II tendon injuries are difficult to treat as the flexor digitorum superficialis and flexor digitorum profundus tendons are vulnerable to adhesion formation or rupture after primary repair.^1^ Bulky repairs lead to triggering when the repair catches on a pulley. Tendon rupture occurs when excessive stress is placed on the repaired tendon, due to patient nonadherence to postoperative care, or inappropriately forceful hand therapy.^2^ New methods maximize strength of primary repair and eliminate gap formation, allowing for both decreased bulk and early mobilization so as to avoid these poor outcomes.^3^

Appropriate postoperative hand therapy is crucial in regaining flexor function. Stressed tendons heal faster and are stronger, develop fewer adhesions, and have better function than tendons that remain immobilized.^2^ A good rehabilitation program can decrease the postoperative complications of tendon repair without sacrificing future mobility. Common therapy regimens include the Kleinart and Duran protocols. The Kleinart protocol involves the use of elastic bands hooked onto the nail plate for passive flexion with active extension against the resistance of the bands, whereas the Duran protocol involves the patient passively flexing the fingers with no resistance against active extension.^3^

When tendon repairs rupture within 3 weeks of the initial tendonorraphy, the tendon can be readvanced and repaired primarily.^4^ However, after 3 weeks, scar adhesions leading to sheath stiffening and collapse make readvancement difficult. In this situation, a 2-stage reconstruction involving tendon grafting must be considered. Silicone Hunter rods can be used in the first stage while the pulley is repaired and a new sheath reforms around the rod, at which point a tendon graft can replace the temporary rod in the second stage of repair.^5^ As in the initial repair, close adherence to a hand therapy protocol in conjunction with a good surgical technique that uses strong core sutures and reduces tendon gapping can reduce the incidence of complications such as ruptures.^6^

Our patient successfully underwent delayed secondary repair of the flexor digitorum profundus tendon and the flexor digitorum superficialis tendons 18 days after initial presentation. However, she failed to return for follow-up on the first postoperative day or participate in hand therapy. She presented 10 days later and was found to have suffered a rupture of her primary repair due to premature removal of the dorsal blocking splint. The patient was subsequently lost to follow-up. This unfortunate case underscores the importance of meticulous surgical technique and patient compliance in postoperative follow-up to obtain optimal results.

## Figures and Tables

**Figure 1:**
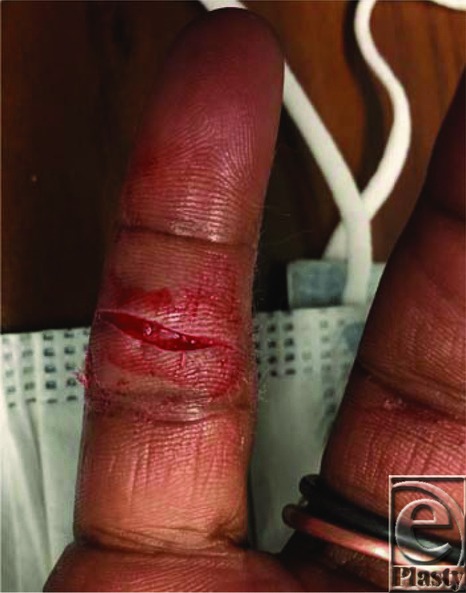
Volar laceration.

**Figure 2:**
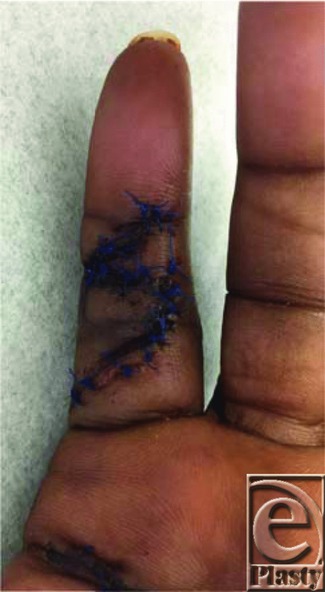
Patient represented after removing splint.

## References

[1] Wilhelmi BJ (2014). Flexor tendon repair. Grabb and Smith's Plastic Surgery.

[2] Momeni A, Grauel E, Chang J (2009). Complications after flexor tendon injuries. Hand Clin.

[3] Lutsky KF, Giang EL, Matzon JL (2015). Flexor tendon injury, repair and rehabilitation. Orthop Clin N Am.

[4] Pulos N, Bozentka DJ (2015). Management of complications of flexor tendon injuries. Hand Clin.

[5] Freilich AM, Chhabra AB (2007). Secondary flexor tendon reconstruction, a review. J Hand Surg.

[6] Neumeister MW, Amalfi A, Neumeister E (2014). Evidence-based medicine: flexor tendon repair. Plast Reconstr Surg.

